# Rethinking animal models of sepsis – working towards improved clinical translation whilst integrating the 3Rs

**DOI:** 10.1042/CS20200679

**Published:** 2020-07-10

**Authors:** Manasi Nandi, Simon K. Jackson, Duncan Macrae, Manu Shankar-Hari, Jordi L. Tremoleda, Elliot Lilley

**Affiliations:** 1 School of Cancer and Pharmaceutical Science, Faculty of Life Sciences and Medicine, King’s College London, London, U.K.; 2 Faculty of Medicine and Dentistry, Institute of Translational and Stratified Medicine, School of Biomedical Sciences, University of Plymouth, Plymouth, U.K.; 3 National Heart and Lung Institute, Imperial College London and Royal Brompton and Harefield NHS Foundation Trust, London, U.K.; 4 School of Immunology and Microbial Sciences, King's College London and Guy’s and St Thomas’ NHS Foundation Trust, London, U.K.; 5 Blizard Institute, Barts and the London School of Medicine and Dentistry, Queen Mary University of London, London, U.K.; 6 Research Animals Department, RSPCA, Southwater, U.K.

**Keywords:** sepsis, research animals, 3Rs, Clinical translation, construct validity, mechanistic

## Abstract

Sepsis is a major worldwide healthcare issue with unmet clinical need. Despite extensive animal research in this area, successful clinical translation has been largely unsuccessful.

We propose one reason for this is that, sometimes, the experimental question is misdirected or unrealistic expectations are being made of the animal model.

As sepsis models can lead to a rapid and substantial suffering – it is essential that we continually review experimental approaches and undertake a full harm:benefit impact assessment for each study. In some instances, this may require refinement of existing sepsis models. In other cases, it may be replacement to a different experimental system altogether, answering a mechanistic question whilst aligning with the principles of reduction, refinement and replacement (3Rs).

We discuss making better use of patient data to identify potentially useful therapeutic targets which can subsequently be validated in preclinical systems. This may be achieved through greater use of construct validity models, from which mechanistic conclusions are drawn. We argue that such models could provide equally useful scientific data as face validity models, but with an improved 3Rs impact. Indeed, construct validity models may not require sepsis to be modelled, *per se*. We propose that approaches that could support and refine clinical translation of research findings, whilst reducing the overall welfare burden on research animals.

## Introduction

### What is the clinical need?

Sepsis arises from a dysregulated host response to a microbial infection and can lead to septic shock. Patients with this syndrome experience circulatory, cellular and metabolic abnormalities which can lead to life-threatening organ dysfunction [1]. Meaningful global mortality estimates are difficult to ascertain, and research is often limited to reporting from high-income countries, but it remains a leading cause of deaths and critical illness worldwide [2,3]. Short-term mortality has been estimated at 45–50% and half of those that go on to survive may have subsequent long-term decline with approximately one in six, dying within a year [4–6].

It has been argued that **earlier diagnosis** of sepsis will have the greatest impact on patient outcome and reduce the economic burden on the healthcare provider [7,8]. Earlier diagnosis allows clinicians to treat and manage the underlying infection using standard approaches (including source control and antimicrobial therapy) and to anticipate or manage cardiovascular instability and other evolving organ dysfunction [9,10]. The later the diagnosis, the more complex clinical management becomes, with a rapid rise in mortality risk and ongoing complications for survivors [11].

Another argument highlighted in this context is that knowledge of different sensitive and specific serological and physiological **biomarkers** and the development of **early warning scores** and multi-biomarker algorithms, may aid in identifying and stratifying patients and assist clinicians in planning investigation and treatment, and in particular identifying those at the highest risk [6,12–16]. In addition, understanding certain known or yet to be discovered biomarkers could lead to the identification of therapeutically useful **new targets or diagnostic approaches**, which may need to be validated using non-clinical systems.

Clinical manifestations of sepsis are protean, risk of adverse outcome and response to treatments also vary between patients. The trajectory of illness progression and recovery from sepsis in humans has also not been well studied. Thus, given that there is no single trajectory of sepsis progression, treatment or resolution in humans – there are significant challenges with meaningfully modelling the clinical syndrome in research animals.

In the Table 1, we summarise certain current clinical needs and research approaches that could address those needs and indicate where research animal data may still be required.

**Table 1:** Current clinical need and where research animals fit in

Clinical need	Research approach	Does this necessitate the use of research animals?
Effective antimicrobial agents Immunological modulators Agents to treat common symptoms with improved efficacy/safety profile e.g. coagulopathy, myocardial dysfunction, delirium	Demonstration of on-target engagement and safety. Identification of detailed biomarkers associated with on-target efficacy and/or safety – to inform future conduct of clinical trials	Yes – where there is no alternative. We propose studies should be mechanistic in nature (see Box 1)
As above	Drug repurposing	Less likely – regulatory safety pharmacology, toxicology and pharmacokinetic studies completed
Biomarkers corresponding to beneficial/detrimental responses to existing treatment Biomarkers facilitating diagnosis Biomarkers/modifiable elements facilitating patient stratification into risk groups	Information gained from better use of patient health record data	Unlikely

### What is the preclinical problem?

In the context of preclinical sepsis research, there has been a poor clinical translation of therapeutic entities validated in animal models of sepsis. Blame for the widely reported translational ‘valley of death’ [17] is often placed at the feet of preclinical researchers for using flawed animal models or for inappropriate experimental design and/or reporting. Whilst these are valid arguments, we propose that another reason for lack of translational impact is that, in some cases, current preclinical animal models are not capable of answering the experimental question being asked. Where the question is ‘will my putative treatment provide clinical benefit to sepsis patients’, we would argue that most current animal models cannot provide a meaningful, predictive answer.

Translational paradigms of ‘bench to bedside strategy’ may have been inappropriately applied – particularly when aiming to model complex multisystem conditions like sepsis. Bedside to bench and back – where clinical data help to define specific targets or probe specific mechanisms, may prove more fruitful.

From a research perspective, it is imperative that preclinical scientists engage with clinicians to understand the clinical need. Additionally, wider interdisciplinary collaboration (bioengineering, epidemiology, statistics) can ensure that:

- the right targets/strategies/pathways are identified and studied based on clinical precedence through available human data;
- the most appropriate experimental systems and endpoints are being used to validate the value of investigational therapeutic entities and biomarkers;
- new and improved data acquisition and analysis/statistical techniques are being used to improve quality of results, whilst impacting positively on research animals;
- realistic interpretations are being made of the preclinical data, avoiding ‘over translation’.

### How animals are currently used in sepsis research – ethical and translational issues

There are two main categories under which research animals are used in the context of sepsis research:

1. to understand the mechanism of sepsis syndrome progression and identify involvement and changes in physiological, molecular and/or cellular pathways, and/or
2. to assess the efficacy/safety/pharmacokinetics of therapeutic targets/agents for proof of concept or regulatory studies.

To date, sepsis and septic shock modelling methods have either involved administration of an inflammatory trigger (e.g. endotoxin), a microbial trigger (e.g. bacterial or peritonitis) or co-morbidity models (e.g. trauma plus infection). All these models have the potential to cause significant suffering, if conducted in conscious animals.

Justification for the use of any particular model must always have a clear scientific rationale and this by extension, should include a full **harm:benefit impact assessment**. In this context, ‘harm’ refers to the experience of the animal during the experiment (or indeed over the lifetime of the animal if the legislative framework requires this) whilst ‘benefit’ relates to the scientific value of data generated from the study.

Of key importance is the fact that **animal models of sepsis can lead to mortality**, which is still used today as an experimental endpoint. Given the historically poor translation of animal models of sepsis, use of **mortality as an endpoint is becoming increasingly difficult to ethically and scientifically justify** from a harm:benefit perspective. However, progress is being made here, for example the *Galleria mellonella* moth larvae could represent a replacement for mammalian models where survival endpoints are needed – providing a rapid efficacy screening tool for novel antimicrobial agents [18].

Whilst efforts are being made to reach consensus on what constitutes experimental sepsis (in mammals) in order to standardise the scientific literature [19], it is not the purpose of the present paper to critique different ‘sepsis syndrome’ modelling approaches. Furthermore, recommendations have already been published on how to optimise animal welfare within such models [19, 20] and will not be discussed in detail. However, in the present paper we will consider how we might rethink our use of animal systems in sepsis research to reduce the translation gap and by extension impact positively on the application of the reduction, refinement and replacement (3Rs) principles.

### Why do preclinical sepsis models translate poorly?

It is well recognised that no single animal model can recapitulate the complex and varied clinical manifestations of the sepsis syndrome and there are clear differences between what is modelled in research animals and what is seen in patients. Nevertheless, we argue that as long as a harm:benefit analysis has been conducted and that **realistic interpretations** are being made of the data, such experiments can still be of scientific value, especially with regard to furthering the understanding of the underlying pathophysiology and identification and validation of novel therapeutic and diagnostic approaches.

Common criticisms of many animal models of disease, include the use of **single sex, young** animal cohorts, typically influenced by cost, time and infrastructure limitations as well as a desire to limit total animal numbers in line with the 3Rs. Introduction of experimental bias through **lack of randomisation, blinding** or **inappropriate statistical analysis** is also commonly criticised in many animal models (and research in general) but reassuringly, initiatives in education and transparency coupled with planning and reporting guidelines from scientific journals and research funders, may lead to improvements [21–25]

**Take home message**

Experimental sepsis research must be designed, conducted and reported in such a way to minimise bias and to maximise the potential for replication.

Criticism of sepsis models *per se*, have focussed on questionable experimental **time courses**, including the duration of the ‘sepsis syndrome’, choice of endpoints or the **length of follow up in survivors** – which do not necessarily correspond to the clinical situation. It has been argued that the duration of follow-up is often too short and may not capture the more persistent changes in the inflammatory response that ensue in patients [26, 27]. However, performing longer term sepsis studies in animals has the potential to increase the welfare burden and this requires careful consideration of harm vs. benefit. Indeed, it is still not known whether extending such studies in animals will improve our understanding of, or better predict, longer term outcomes in patients.

Studies have also been criticised for **timing of interventions**. Here the research question and the context in which the data are interpreted is key. One argument is that pre-treatment with a new therapeutic entity does not reflect the projected clinical use, where such agents would be administered after symptom development. However, ‘pre-treatment’ studies may be scientifically valid, if a ***mechanistic* research question** is being asked and ***mechanistic* conclusions** are drawn. Indeed, for studies involving genetically altered animals, these would typically have a pre-existing change in the gene product expression (e.g. gene knockout). This approach is scientifically valid as a proof of concept study to demonstrate the relationship between a gene product and its impact on one or more biological pathways that may become dysregulated during inflammation/infection. **It does not, however, provide direct evidence as to whether the target is therapeutically useful in sepsis patients, as the study was not designed to test this**. This highlights the onus on scientists to carefully define their experimental question and accurately interpret the results. Similarly, journal reviewers and editors should ensure data are interpreted in the context of the mechanistic question, avoiding over optimistic data interpretation or ‘over translation’.

Another criticism is the lack of routine supportive therapies such as antimicrobial, fluid resuscitation and other supportive interventions that are not routinely incorporated into models. We will discuss this issue later in a later section ‘***Modelling sepsis in animals – mimicking the clinical setting***’.

**Take home message**

Harm:benefit impact assessment and full scientific justification should be at the forefront of any new studies involving research animals. Data should be interpreted in the context of what the model can deliver, avoiding over-optimistic translation.

### Homogeneity vs. heterogeneity; face vs. construct validity

A key difference between the hospital and laboratory setting is the greater **homogeneity of experimental animal** systems compared with the highly **heterogeneous clinical population**. Homogeneity can be considered an experimental necessity as it enables an investigator to assess the difference between two treatment groups – carefully controlling confounding factors. However, the expected lower variability in inter-animal responses could lead to a relatively modest effect size to be deemed statistically significant. In turn, this could lead to over-interpretation and misdirected prediction of the potential clinical impacts [28].

In contrast, there is extremely high patient heterogeneity in the clinical setting, where sepsis affects neonates through to elderly, co-morbid patients. Differences in the patient demographics, infection source(s), route of entry etc., all lead to complex variability in both the syndrome progression and in responses to clinical interventions. The ability to detect clinically meaningful effects of new therapeutic entities, on the background of such variability, becomes highly challenging. Arguments have been made as to why such clinical trials fail so frequently and to perhaps rethink clinical trial design, including improved stratification of trial participants into phenotypic groups, more detailed biomarkers as endpoints and the use of adaptive trial design [29,30].

It has been suggested that more heterogeneous animal models, e.g. using outbred strains [31], modelling co-morbidities or introducing changes in husbandry moving away from a highly controlled pathogen-free environment [32,33], may address issues of reducing homogeneity in animal studies. However, this could lead to ‘**uncontrolled heterogeneity**’ which could make experimental interpretation and clinical translation, more difficult. Methods to **systematically introduce heterogeneity** [34] may be helpful but would require infrastructure changes to facilitate this.

Whilst on one level, making experimental systems ‘look’ more like the heterogeneous clinical population (**face validity**), may appear sensible, it may not necessarily improve clinical translatability of the results. It would also likely necessitate larger groups of animals in order to demonstrate meaningful effect sizes, going against the principle of the ‘R’ of reduction.

We propose that an alternative approach would be to ask more ***mechanistic* questions** (**construct validity**) of the experimental systems [35]. For example if a plasma biomarker is essential, it may be sufficient to show target engagement of a test compound, as reflected by a change in that biomarker coupled with test compound plasma concentration levels, as opposed to demonstrating an impact of that compound on sepsis syndrome progression. This approach avoids some of the predictive validity issues associated with disease models, with the potential to reduce the welfare burden for animals [35].

Indeed, it has been argued that **target engagement, exposure at the site of interest**, and some indication of **pharmacological activity** (described as the ‘three pillars of survival’) is adequate for Phase II clinical trials [36]. We would argue that preclinical assessment of efficacy in animals could be approached in the same way – especially given the historically poor clinical translation of sepsis model data.

Research to further our fundamental understanding of the dysregulated immune response that occurs in sepsis is still needed – and this may still require research animals. However, we would argue that these studies should also have a mechanistic focus and results should be interpreted within this context. Indeed, the fundamental immunological and molecular signalling differences between humans and common laboratory animals, highlight the experimental challenges [37,38]. Such insights might be better gleaned from analysis of increasingly available clinical electronic health record data.

**Take home message: Construct validity and the 3Rs**

Focussing on construct rather than face validity, has the same potential to increase clinical translation, but with reduced levels of animal suffering.

In summary, mechanistic studies may still utilise existing models of sepsis, but with more careful consideration of time courses, biomarker measurements and avoidance of death as an endpoint. This approach could still address important scientific questions whilst impacting positively on the predictive validity of the model and welfare of the research animal [39] (Box 1).

Box 1 **A comparison of disease models (face validity) with mechanistic models (construct validity) and their potential scientific and 3Rs values**

Mechanistic models
  A major theme of this review is the proposal that preclinical sepsis research can be better served using mechanism-based models rather than phenotypic-disease models. To clarify what we mean by this, let us give our definition of these two approaches.
Disease model
  An *in vivo* disease model is one where, via surgical, chemical, biological or genetic intervention, an animal displays a phenotype that looks like (has high face validity) one or many aspects of the equivalent human disease. Such a model will typically use a disease score or a surrogate to a human clinical outcome measure (e.g. mortality in sepsis) as a primary experimental endpoint. For example, the rat collagen-induced arthritis model [40] involves immunisation with type II collagen which can lead to severe arthritis with bone matrix resorption, considerable soft tissue swelling, periosteal new bone formation and bone erosion.
Mechanistic model
  A mechanistic model (with high construct validity) is optimised to provide a robust signal-to-noise ratio for engagement (activation or inhibition) of a specific target mechanism. This may or may not involve an animal, but the advantage of an *in vivo* mechanistic model is the possibility of concurrent measurement of the plasma level of pharmacological interventions or biomarkers. Such a model can share similar induction methodology as a disease model, but importantly has specific mechanism-based endpoints rather than phenotypic endpoints, for example the concentration of a specific plasma biomarker.
  Alternatively, the mechanistic model may have little similarity to the disease of interest, providing the mechanism being studied is the same, and the signal-to-noise ratio is optimised. For example, a drug discovery programme based on a hypothesis (derived from human clinical genomic data) that inhibition of a specific cytokine would be beneficial in rheumatoid arthritis. The key information required for progression of a new drug into clinical development is adequate target engagement at a given plasma level (how much drug is required to inhibit the action of the cytokine). This information can be obtained from any *in vivo* model where the biological activity of the cytokine can be measured, and this does not have to be a model of arthritis *per se*.
  For both types of model, a full harm:benefit impact assessment should ensure the appropriate animal model with lowest welfare concern for highest scientific value, is chosen.

## What needs to change? Part 1 - Focus on improving mechanistic insight into sepsis pathophysiology

Isolating particular features of the clinical condition may help to develop more mechanistic models which may prove useful in answering discrete biological questions. Indeed, the sepsis syndrome can be broken down into a series of key features [1]. We will explore some of these in more detail below.

### Immune dysfunction

The central importance of the immune system in sepsis has prompted the search for specific therapies targeting the immune response. However, despite considerable research effort with experimental models and numerous clinical trials, there are no current immune-based treatments for sepsis, in routine clinical use. This failure highlights the disconnect between existing experimental models of sepsis and the complex disease in patients. In particular, the lack of translation between highly inbred mouse strains with specific infective foci and the considerable heterogeneity of the sepsis-induced immune response both between patients and within individuals over time [39,41,42]. Such heterogeneity suggests that any single treatment strategy will not succeed and that a personalised medicine approach is required. Therefore, it is likely that the success of future trials of sepsis therapies will rely on precision medicine approaches, with a focus on ascertaining the underlying immune responses in patients. Mechanistic models of the immune dysfunction in sepsis are thus required to support the development of new immune-targeting therapies and to identify biomarkers to monitor the immune status and guide treatment.

At present, immune status is derived from plasma markers or expression of molecules on circulating blood cells. However, the immune response is highly compartmentalised and varies over time and the phenotype of circulating blood cells may not be the best indicator of current immune status. Existing animal models of sepsis often show enhanced cytokine production despite having profound leukopenia suggesting blood immune cells have a limited role in producing inflammatory mediators for host defence [43]. Moreover, murine experiments have shown that blood and spleen leukocytes become hyporesponsive during endotoxaemia or caecal ligation and puncture-induced sepsis (i.e. tolerance) [44] but the functions of macrophages from different tissues and organs was found to be either unaffected or primed [45,46]. In healthy volunteers given endotoxin, alveolar macrophages are primed for increased inflammatory cytokine production whereas blood monocytes are shown to be profoundly immunotolerant [47]. These and other findings in human models of endotoxaemia suggest that compartments other than blood, shape the immunosuppression in sepsis [47]. Nevertheless, the ‘tolerance’ induced by endotoxin in human volunteers shows many similarities to sepsis-induced immunoparalysis including decreased cytokine production by circulating leukocytes. Endotoxin tolerance has therefore been used as a model to investigate treatments for sepsis-induced immunoparalysis, both *in vitro* [48] and *in vivo* in humans [49]. In contrast, however, animal studies have shown that despite a reduced immune response, pathogen clearance and survival (after live bacterial challenge) were enhanced in mice pretreated with LPS or other TLR ligands [50]. It is unknown whether the same pathogen clearance effect would be seen in humans and this could be studied in future trials. New experimental models that report on the tissue immune response are thus urgently required to improve our understanding of the mechanisms of the immunological response during sepsis and to guide the use of immune-modulating therapies.

Platelets are another blood immune cell implicated in the excessive inflammatory host response during sepsis, which can be both pro- and anti-inflammatory/thrombotic in nature. Correlations have been observed in patients, between platelet number and mortality rates with low platelet count (thrombocytopaenia) correlating with poor outcome [51–53]. This has prompted studies to evaluate the potential benefits of platelet therapy in sepsis [54]. Therefore, mechanistic models are required to provide a better understanding of the role of platelets in sepsis.

A more accurate picture of the *in vivo* immune response will also likely require a panel of biomarkers that reflect the immune response in different compartments and at different times. Many current biomarkers, such as circulatory inflammatory cytokines are prognostic rather than diagnostic in value. Others have a high sensitivity but low specificity (CRP) and may not distinguish between hyperinflammatory states and immunoparalysis (procalcitonin). Potential markers of immunoparalysis are proposed from patient studies including expression of inhibitory receptors like programmed death-1 (PD-1), its ligand PD-L1 and cytotoxic T lymphocyte antigen-4 (CTLA-4) [55]. In addition, suppressor lymphocyte populations (T-regs) have been linked to patients with immunoparalysis [56].

Identifying and understanding the causes of hyperinflammation and immunoparalysis in sepsis patients is the key to developing effective immune-based therapies. Interestingly, meta-analysis and **post hoc analyses of data from the many ‘failed’ trials of anti-inflammatory agents** have revealed that such therapies may have been **effective in subgroups of patients** [57]. This again highlights the need to stratify sepsis patients into defined subgroups, so they receive the most appropriate therapy.

### Systems biology

Advances in our mechanistic understanding of susceptibility to adverse sepsis outcomes may come from human patient studies and replace traditional experimental animal models. The development of translational high-throughput methods and analysis tools has allowed the identification of distinct subgroups based on host response to sepsis. These subgroups or ‘endotypes’ will facilitate patient stratification, to identify the most effective therapies. Transcriptomic profiling of sepsis patients has defined different sepsis response types which could be linked to immune suppression, metabolic derangements and 14-day mortality [58]. Significantly, these response endotypes could be accurately predicted from seven genes. Interestingly, a different study identified the same endotypes in faecal peritonitis patients, with similar outcomes [59]. A comprehensive transcriptomic profile of 700 sepsis patients revealed three subgroups – the one with the highest mortality and reduced adaptive immunity, corresponding to the most susceptible group defined in the earlier studies [60]. Such profiling should translate into identifying patients who would benefit from immunostimulatory therapy.

### Trained immunity

Observations in mammalian models of vaccination which found that protection from reinfection occurred independently of T and B lymphocytes led to the hypothesis that innate immunity can display characteristics of adaptive immunity in response to pathogens or their products, and has been coined as ‘trained immunity’. The discovery of trained immunity allows the opportunity to develop models to evaluate the effects of immune training on sequelae and outcomes in polymicrobial sepsis, and the impact of immune training in the prevention and/or treatment of sepsis-induced immune paralysis and organ injury [61]. Interestingly, trained immunity also provides a mechanistic link between sepsis and atherosclerosis that has been observed in animal models of sepsis [62].

### Metabolic switching

Growing evidence supports the view that metabolic changes underlie immune responses including the immune cell phenotype in sepsis [63]. Indeed, LPS activation of TLR4 signalling pathways is associated with switching to glycolytic metabolism in the activation of macrophages. These changes in glucose metabolism mirror the immune responses seen in septic patients with a switch from a more transient hypermetabolic anabolic state to a hypometabolic state in cell, animal and human models of sepsis. Sirtuins (SIRT) play a crucial role in this metabolic switch coincident with the switch from hyper- to hypo-inflammatory response and animal studies show the mechanistic benefit of targeting sirtuin-1 in sepsis [64, 65].

The HIF signalling pathway has gained significant importance as a possible immunometabolic switch and HIF has been proposed as a sepsis biomarker and therapeutic target [66]. Indeed, it was recently shown that myeloid HIF-1 plays an acute time-dependent role in regulating the peripheral glycolytic response [67]. Mechanistic studies using human cells from patients and valid animal models, will provide a more clinically relevant understanding of the metabolic alterations which occur in innate immune cells and the role of HIF signalling [66].

### The inflammasome

Further improvements to the translational value of sepsis models will likely come from a better understanding of the mechanisms of innate immune responses to infection. One such area of intense research is the inflammasome. The best characterised is the NLRP3 inflammasome, that also contains adapter protein apoptosis-associated speck-like protein (ASC) and procaspase-1 that activates the inflammatory cytokines, IL-1b and Il-18 and induces a form of cell death known as pyroptosis [68]. Recent studies demonstrate that NLRP3 inflammasome-related molecules (e.g., NLRP3 and ASC) are critically involved in the acute inflammation and tissue injury involved in the pathogenesis of CLP-induced polymicrobial sepsis. Inflammasome dysfunction is a recognised feature of sepsis-induced hyperresponsiveness [69] and growing evidence suggests that inflammasomes play a critical role in bacterial infections [70,71]. NLRP3 inflammasomes interact with many molecules associated with a variety of conditions. For example, an overactivated NLRP3 inflammasome in immune cells is associated with both obesity and insulin resistance and T2DM. Therefore, it is possible that pre-existing dysregulated inflammasome function may predispose patients to sepsis, from an infection that could be overcome in otherwise healthy persons [72]. Drugs designed to target NLRP3 inflammasome activation show promise in treating NLRP3-dependent inflammatory diseases including sepsis in animal models [73,74].

### The human model of the immune response in sepsis

The immune response in sepsis involves the complex interplay of thousands of genes in a ‘genomic storm’ [75]. Such complexity and heterogeneity suggest that a single animal model may not be appropriate, and any single sepsis therapy, unachievable. Research in sepsis is currently shifting away from models that poorly reflect the human condition, towards a mechanistic understanding of the complex pathways in the sepsis process. Understanding how these pathways interact, may lead to the identification of common targets that can affect downstream signalling events.

Development of human models of the immune response will better aid the translation of fundamental mechanistic studies. These include *ex vivo* and *in vitro* studies of human immune cells directly, including those from blood and tissues. Studies using peripheral blood are valuable due to the relative ease of sampling, and the potential for future translation in diagnostics and immunotherapies. Human challenge models, in which healthy subjects are infected with a controlled amount of microorganism or their components, will also provide relevant mechanistic data. Furthermore, new systems biology tools will allow the high throughput of human-derived data, which has been the advantage of current animal models [76]. Development of more sophisticated models allowing more detailed knowledge of the human immune system in sepsis, will enable the identification of new immune mechanisms that can be translated into new diagnostic and therapeutic practices.

**Take home message: Immunological and metabolic**

As our knowledge of the innate immune responses in sepsis increases, new therapeutic targets and new potential biomarkers to measure sepsis progression and response to therapy are emerging. These include metabolic markers that underlie trained immunity and immune cell activation and biomarkers of inflammasome activation which could be mechanistically investigated in preclinical models. As tools and technologies for systems biology and high-throughput screening advance, there can be an increased use of humans and human-derived tissue for sepsis research models. Development of more sophisticated models will enable the identification of new immune mechanisms that can be translated into new diagnostic and therapeutic practices.

## Circulatory

Circulatory disturbances form a hallmark of sepsis (septic shock) and circulatory shock is associated with increased mortality rates [77]. Given the lack of clinically available immunomodulatory therapies, current patient treatment typically focusses on control of the source of infection (draining or releasing any focus where possible), antimicrobial therapy and empirical haemodynamic support.

Clinical recommendations are in place regarding haemodynamic support for patients including fluid, vasopressor and cardiac inotrope administration of well-characterised agents [10]. However, optimising and managing haemodynamic support strategies in sepsis patients is not always straightforward. Nevertheless, it could be argued that a key clinical need, is a mechanism to facilitate optimisation of individual patient dosing regimens for escalation and de-escalation of treatment [78]. Such information could be better identified through retrospective clinical data, rather than from further animal experimentation. Use of retrospective patient data would allow specific regimens and to be linked to patient outcomes and could reveal specific patient signatures that associate with advantageous vs. disadvantageous responses to treatment. The clinical benefit of such signatures would, of course, require validation using a prospective randomised controlled trial (RCT). However, if validated, such information could be consolidated into clinical decision support tools and scoring systems. This type of research will necessitate an interdisciplinary approach to data collection, annotation, analysis and interpretation, but proof of concept studies already exist [79,80].

### Fluid resuscitation

Relative hypovolaemia is common in patients with sepsis and leads to reduced cardiac preload resulting in reduced cardiac output and systemic oxygen delivery. Fluid is administered during resuscitation to replace the fluid deficit to augment cardiac filling and cardiac output. Clinically, fluid resuscitation in patients can be guided by haemodynamic and biochemical variables and there are international recommendations in place [10]. In small animal studies (mice, rats), the continuous assessment of such variables is not as always feasible, but is possible in larger species such as pigs [81]. As with humans, fluid resuscitation regimes have been investigated in animal models with certain regimes improving haemodynamic variables and outcomes [82,83]. Later we will discuss the concept of ‘animal ICUs’ allowing intensive investigation of individual trajectories from anaesthetised, fluid resuscitated, preparations.

Scientists who wish to model hyperdynamic changes in research animals require effective fluid resuscitation. For small lab species such as mice and rats, this can be technically challenging to perform and fluid overload can easily occur, resulting in inadvertent oedema. However, hyperdynamic rodent models do exist [84–86]. From a welfare perspective, high volume resuscitation should be conducted via indwelling vascular access ports [87] which facilitates continuous or multiple infusions. However, intraperitoneal or subcutaneous resuscitation tend to be more commonly performed as single boluses [20]. The varied nature of fluid resuscitation (e.g. crystalloid vs. colloidal, continuous vs. bolus), and the extent to which animals are resuscitated in different labs, can greatly influence commonly measured parameters such as blood pressure, cardiac output and ejection fraction. Furthermore, normalised or delta changes, in such parameters, are often reported in the literature, making it challenging for the scientific community to have a grasp of the absolute degree of change anticipated within a model. Recommendations for the nature of resuscitation have been generated and this may assist in standardising how such experiments are conducted [88]. Whether this improves clinical translation of new therapeutic entities, still remains to be determined.

### Cardiac and circulatory disturbances – *in vivo* and *in vitro*

One of the benefits of preclinical sepsis experimentation, is that the exact time of the infection (t = 0) is known, allowing time-dependent changes to be accurately mapped. **Cardiac and circulatory disturbances** are a hallmark feature of many animal models of sepsis, and mechanistic studies may involve investigating the effects of gene product modifications or new therapeutic entities on the trajectory of cardiovascular dysfunction and tissue perfusion, following infection. Depending on the research question, such studies could be conducted in either **conscious or anaesthetised animals**.

Animal models of sepsis or endotoxaemia have been shown to display many cardiovascular disturbances that correspond to those seen in patients and human volunteers [89–91]. These include tachycardia, tachypnoea, cardiac dysfunction [92], vasodilatation and hypotension [93,94], pulmonary hypertension [95] increased incidence of arrythmias [96], microcirculatory disturbances and time-dependent changes myocardial function and regional tissue perfusion, oxygenation and end organ damage [97,98]. There are however certain fundamental differences between humans and common lab animals, such as non-shivering thermogenesis, observed in mice [99]. Whilst many concerns have been raised as to the translatability of murine sepsis models, this is still a commonly used species for mechanistic studies, given the relative ease of breeding, genetic alteration and lower experimental and infrastructure costs.

Importantly, as discussed earlier, it may not always be necessary to model sepsis *per se* and, where possible, those mechanistic models which pose the lowest welfare burden should be used. At a cellular level, it has been established that microvascular endothelial damage can be aggravated by inflammatory mediators, generated following a host response [100]. It is technically challenging to isolate cellular effects from whole animal systems, although endothelial-specific gene modification and microvascular perfusion measurements are feasible and may provide useful information.

However, advances have been made with *in vitro* replacement models, such as organ-on-chip systems which might help scientists understand endothelial and smooth muscle cell functionality and interactions in mechanistic models of inflammation and could provide a surrogate system to understand gene modification or pharmacological effects [101].

### Cardiovascular monitoring technologies that support refinement

Most routine cardiovascular clinical measures can be quantified without necessitating significant restraint or human intervention, thereby reducing research animal stress responses. These include blood pressure, temperature, ECG, glucose and locomotion radiotelemetry, using implantable devices that enable real-time monitoring of animals, in their home cages. Solitary isolation can be limited via co-housing with unimplanted animals or with implanted animals transmitting data at a different frequency [102]. Telemetry probe implantation surgery does pose a welfare burden, with animals typically requiring 7–10 days recovery time, before readings can be taken. Non-invasive echocardiography can capture more nuanced information about cardiac function and can also be conducted in telemetered animals. Echocardiography is typically conducted under anaesthesia so repeated measures are possible, but this can become challenging, especially in the later stages of the sepsis syndrome, when animals are more compromised and physical restraint/anaesthesia can increase the welfare burden. Certain measures can also be acquired under terminal anaesthesia or post-mortem, such as blood pressure via direct arterial cannulation, tissue oxygenation, microcirculatory perfusion in specific beds, and serological and histological measures pertaining to end organ function. In depth assessment of lung function measurements using technologies such as the FlexiVent (SIREQ) can also be used under terminal anaesthesia for the assessment of sepsis-induced acute lung injury.

Many of these real-time monitoring technologies generate high fidelity data, much of which is typically underused. Interdisciplinary collaboration with bioengineering, statistics and mathematics can help to make more of the data acquired. For example, the extraction of heart rate variability or other detailed cardiovascular changes [103–107] can be generated from preclinical blood pressure or ECG waveforms and can be combined with inflammatory biomarkers, to build a more detailed picture of the physiological status of the animal. Reassuringly, such interdisciplinary analytical approaches have already led to the successful development of early alert systems in patients [108].

In summary, with careful incorporation of more humane endpoints, these experimental techniques when used individually or in combination, generate quantitative measures/biomarkers which can allow discrete mechanistic questions to be answered. In turn, the knowledge of such biomarkers could improve future clinical studies identifying more detail measures pertaining to efficacy and safety, to be incorporated as clinical trial endpoints. We propose that such quantitative measures, acquired at specific time points, better fulfil the harm:benefit assessment, compared with mortality endpoint models.

**Take home message: Circulatory disturbances in sepsis**

Animal models may still be required to validate safety and efficacy of new therapeutic entities that target the cardiovascular system. Several minimally invasive technologies and *in vitro* models are available which can help reduce, refine and replace animal experiments. Real-time monitoring technologies can encourage use of scientifically valuable, humane endpoints, whilst avoiding mortality endpoints. Such detailed endpoints could also usefully be incorporated into future clinical studies.

## What needs to change? Part 2 – Better use of clinical data

### Preclinical outcomes – clinical validation: bridging the gap (valley of death?)

We propose that preclinical research and development in sepsis needs to ‘re-tool’, to recognise that current animal model-driven approaches, lack sufficient predictive validity to affect successful clinical translation. Two things need to change: firstly, research focus needs to be redirected to address the key clinical need for biomarkers that enable early identification of sepsis and effective stratification of patient subtypes for treatment regimens; secondly, more predictive model systems need to be developed.

The 2018 joint report from the Bioindustry Association and the Medicines Discovery Catapult [109] examined the current state of drug discovery in the U.K. and emphasised the highly challenging issue of poor translation. They proposed a radical change in approach that centred on humanising discovery through better use of patient biomarker data and the development of advanced human-based *in vitro* model systems. They argued that effective drug development requires libraries of mechanistic models to be developed that reflect the heterogeneity of human disease. No one would present that this is easy or that it can be achieved overnight, but the ongoing crisis in translation needs to be addressed and a radical change in approach is needed. Critical selection of targets and expected outcomes are crucial, and one cannot just rely on the validity of a one-fits-all preclinical model.

**Figure 1:**
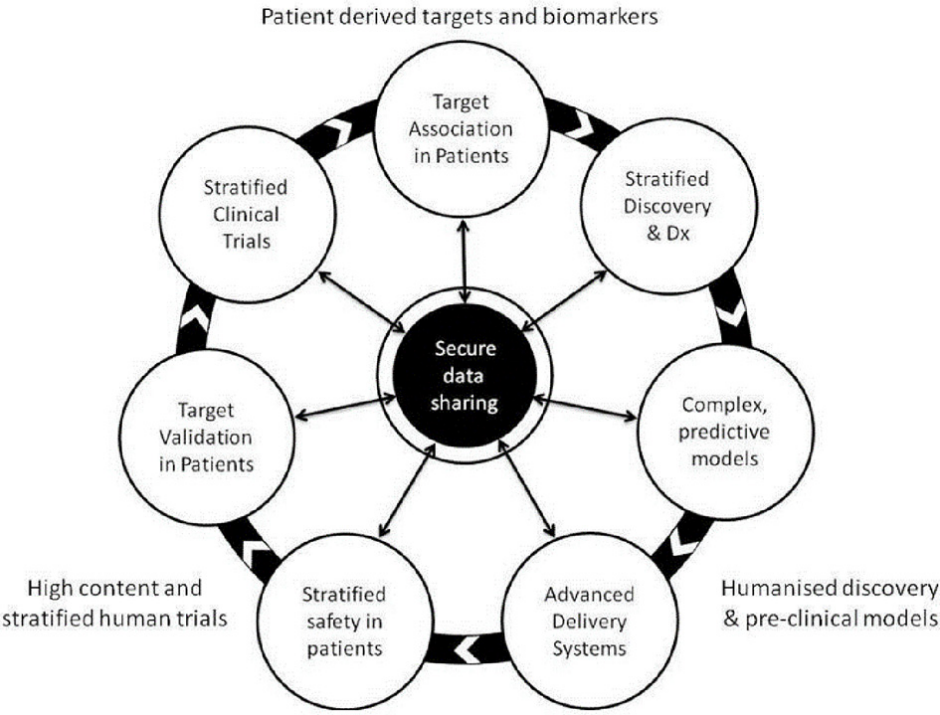
The key features of a ‘humanised’ drug discovery process (Bioindustry Association/Medicines Discovery Catapult Report, U.K. 2018)

Figure 1, taken from the report, nicely illustrates how the three critical components of this approach using patient-derived targets and biomarkers, humanised models and high content stratified clinical trials, come together. Central and critical to this approach is a joined-up mechanism for data sharing, with all stakeholders, working together to achieve translational benefit.

So, how would this work in sepsis research? Clearly, this needs to start with the identification of robust, biomarkers of disease. Currently, vast amounts of data are collected from patients and this needs to be standardised, collated, appropriately labelled, deidentified and analysed; not simply biomarker data but treatment and outcome data too. Additional approaches such as genome-wide association studies (GWASs) need to be applied, as well as any new emerging technologies, and together these can help better identify druggable targets [110,111]. Unsupervised machine learning approaches, can help identify patient subgroups through clustering patterns identified from multiple input variables. We need to paint a comprehensive picture of each patient from their initial presentation through diagnosis, treatment and long-term outcome – including indices of quality of life. Progress is already being made in this area with the development of early warning scores derived from annotated health records [112–114,16,79] but systems like this need to be implemented across the whole healthcare network as part of an integrated data sciences approach. Such a shift in approach will require significant investment and we, perhaps provocatively, suggest that some funding currently directed towards use of existing animal models of sepsis could be redirected to cover this.

Where animals may still be required would be the identification of detailed biomarkers pertaining to on-target and off-target effects of new therapeutic entities. Joined-up research studies which demonstrate the clinical relevance of specific animal models will become increasing possible with the rise in available clinical data [115]. Through mechanistic investigation, such biomarkers could be incorporated into clinical trials to provide more detailed endpoints, which may address the high risk of failure in reaching primary endpoints such as mortality. Importantly, such preclinical investigations may not necessarily require sepsis models.

Key points which may expedite identification and clinical assessment of new therapeutic entities:

1. Clinically relevant targets should be identified in patients (using e.g. GWAS) to ascertain genotype:phenotype associations.
2. Druggability of such targets can be ascertained from existing compound/structural data.
3. If a relevant correlation is established, investigate the target mechanistically, as described, to support development of new compounds or identify suitable compounds for repurposing.
4. Conduct RCT, incorporating detailed mechanistic endpoints.

**Take home message: Top-down approach to sepsis research**

A shift of focus, from animal-based approaches to one that places more emphasis on human clinical data will benefit more patients in the short term and identify new therapeutic targets for drug/biomarker development in the longer term. Understanding causal or correlative relationships between new targets/biomarkers and sepsis progression/outcomes would need to be confirmed through systematic investigation clinical and non-clinical experimental approaches.

## What needs to change? Part 3 – developing more predictive preclinical models

We have outlined a proposal to move away from diseased focussed models (face validity) to mechanistic models (construct validity), but this can only work if sepsis mechanisms are better understood. It could be argued that preclinical disease models are needed in order to elucidate these mechanisms, and we recognise that this is a valid argument. We propose that another approach should also be explored; one where biomarkers identified from clinical health record data (outlined above) are used to target the development of new preclinical models, with a purely mechanistic focus. We argue that this will accelerate clinical development, whilst reducing the welfare burden on animals.

**Figure 2:**
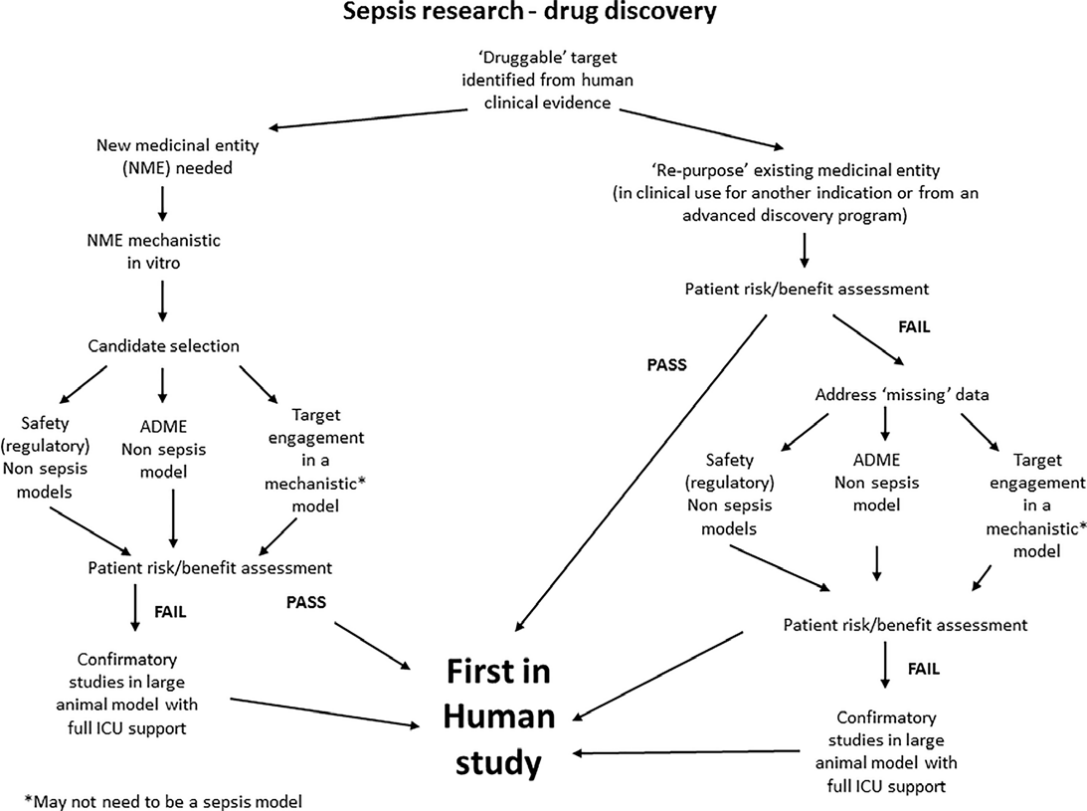
Schematic of proposed pathway for a ‘re-tooled’ drug-discovery programme for a new pharmacological intervention for sepsis

To illustrate our proposed ‘re-tooling’ of sepsis research we have produced a simple scheme (Figure 2). Applied research (where a specific disease target mechanism is known) can involve development of new therapeutic entities or repurposing of existing pharmacological agents, previously developed for a different disease, but which involves the same mechanistic pathway or target. Here, as described above, the key information to enable progression into clinical assessment are:

- target engagement (e.g. efficacy) at a given plasma level (requiring evaluation of the absorption, distribution, metabolism and excretion [ADME] profile), and
- safety (as determined by standard regulatory tests) [116].

We argue that target engagement assessment does not necessitate an animal model of disease (face validity) but rather a model that is optimised to report engagement at the specific target (mechanistic model; construct validity). Indeed, it may not be necessary for this model to resemble sepsis at all. This relies on the target being robust and selected based on clear evidence from clinical data, and should be subject to a patient risk/benefit assessment (where the risk of initiating a first in human trial is assessed based on the currently available preclinical data).

The preclinical model(s) that best enables robust decision making and rapid transition into clinical assessment should be used and these may be very different from those used currently. In this scheme, an animal model of sepsis is only necessary in circumstances where the patient risk/benefit analysis or regulatory bodies, require it. Here, we suggest that the most translational sepsis model is used, e.g. an animal model with full intensive care unit (ICU) support [117].

### Modelling sepsis in animals – mimicking the clinical setting

Due to the complex multisystem pathogenesis of sepsis, preclinical studies modelling the syndrome should ideally aim to replicate those conditions experienced by sepsis patients in ICU. These include pharmacological and supportive interventions (e.g. vasopressors, enteral or parenteral nutrition, resuscitation with intravascular fluids, sedation, mechanical ventilation) with parallel clinical assessments (continuous haemodynamic monitoring, discontinuous serological sampling, blood biochemistry, qualitative clinical observation).

Use of routine supportive clinical interventions, including fluid resuscitation and antimicrobial therapy is considered important when modelling sepsis. However, use of these interventions is not always reported [88]. Hypovolaemia is observed in clinical sepsis and fluid resuscitation is always recommended in animal models for both scientific and welfare reasons [20]. If fluid resuscitation is not performed, it becomes challenging to dissociate scientific outcomes of the sepsis model *per se*, from those that result from hypovolaemia. There are now guidelines in place for resuscitation regimes in the preclinical setting in an effort to standardise this step [88]. Whether adherence to such recommendations impacts positively on clinical translatability, remains to be demonstrated.

Use of antimicrobial therapy in preclinical studies has also been debated. On the one hand, including antimicrobials would better mimic the clinical situation. On the other hand, it may interfere with the model progression, potentially obscuring the elucidation of cellular or molecular mechanisms. The use of antimicrobial therapy has been investigated in numerous animal models [118–120] and there are existing recommendations on how use of antibiotics might improve the clinical translation of preclinical data [88]. However, antimicrobial use must be scientifically justified, and **antibiotic stewardship** should always be prioritised to avoid contributing to antimicrobial resistance development both within and outside, animal research units [121].

These implementations can be challenging when using small laboratory animals, particularly rodents, due to considerable haemodynamic profile differences (very high circulation times and limited blood volumes in comparisons with larger mammals and humans) and complexity of microsurgical interventions. However, adaptation and miniaturisation of monitoring equipment for use in smaller laboratory animals is now making it possible (e.g. specialised mechanical ‘lung-protective’ ventilators that minimise the risk of injury through improvements in tidal volume and positive end-expiratory pressure). However, innate high resilience to ischaemic–reperfusion injury, poor thermogenesis and physiological limitations for repetitive sampling, remain important challenges in rodents. Nevertheless, mechanistic and early-phase discovery studies in rodents should, wherever possible, implement extensive physiological monitoring (particularly blood pressure and body temperature) and fluid support/resuscitation, to better mimic the clinical situation [86].

Due to these challenges, the use of larger animals in such ICU settings, particularly the porcine shock model, has increasingly been used. A larger species allows the use of existing clinical monitoring equipment, repetitive blood sampling (valuable for differential and blood gas analysis) supporting better titration of dosing, in accordance with routine point-of-care readouts and biomarkers. They also mirror the thermogenesis response to stress and the systemic energetic failure associated with septic shock (circulatory shock). Furthermore, there is an increasing need for mid-longer term understanding of the pathophysiology of sepsis and how proposed interventions impact on sepsis patients during their stay in ICU, particularly on the onset and progression of organ dysfunction. Models in which it is possible to perform longer term studies, with full ICU support would therefore be useful. However, there are considerable constraints related to infrastructure and equipment, staffing and funding. In addition, current equipment and protocols make maintaining animals in an ICU setting challenging due to complications related to mechanical ventilation – the maximum duration is ∼15–24 h in rodents, 100 h in pigs, with pigs being particularly susceptible to develop impaired lung function associated with pronounced acute pulmonary hypertension [122,123].

The implementation of preclinical ICU-mimicking studies, with an intensive monitoring and a care programme, are also critically important from an animal welfare perspective. As stated previously, the intrinsically severe nature of many sepsis models, and the impact that this has on the validity and quality of the experimental data raises significant ethical issues. For example, sympatho-adrenal responses to stress result in increased circulating levels of catecholamines which will trigger tachycardia and hypertension, pain will also modulate the HPA axis affecting cortisol and growth hormone release, among others effects on homeostasis [124,125].

Larger animal ICU paradigms also allow for studies to be adaptive in nature, responding to real-time biomarkers and this more closely resembles adaptive approaches in human clinical trial design [126]. However, this is not always possible in smaller rodent models which tend to use predefined time courses and intervention protocols and where repeat blood samples will be limited by small circulating volume.

The establishment of comprehensive, clinically relevant care/welfare and experimental outcomes remains a significant challenge in sepsis modelling, with technical as well as infrastructure, staff training and cost limitations. However ICU monitoring settings will likely support better indicators of disease morbidity and implementation of humane endpoints.

**Take home message: Mimicking the clinical setting**

Current preclinical approaches are not delivering for sepsis patients. We propose the concept that a single animal model could be the ‘gold standard’ predictor of clinical success should be abandoned. Mechanistic models, derived from human clinical data, with a focus on target engagement rather than clinical phenotype should be more predictive. If disease (face validity) models are required, these should mimic human clinical ICU settings as closely as possible.

## Summary

Sepsis can be devastating for patients and their families, even those that survive can experience long-term health issues. Clearly, research is needed to find new and better ways to combat this; we do not dispute this. In the present paper, we have described some of the reasons why current research approaches have mostly failed to translate into clinical benefit and offer a deliberately provocative alternative approach. We suggest that sepsis research funding could, in the short term, be redirected towards better characterisation of human patient data linked to outcome such as proteomics/metabolomics and other biomarker studies in patients. We suggest that these data will identify new therapeutic targets for preclinical research or identify more sensitive and specific diagnostics or biomarkers for patient stratification. Where appropriate, targets can be tested using mechanistic animal models, which may or may not be based on traditional animal models of sepsis. Where a preclinical proof of concept efficacy study is justified, we propose that large animal models with full ICU support may have the greatest translational potential. Whilst we do not dispute the value of animal models in medical research, we assert that the value of any model is diminished if it is stretched beyond the point where it is fit for purpose. A harm:benefit assessment should always be conducted prior to any study and based on pre-existing clinical or preclinical evidence. We hope that this review stimulates debate and encourages new collaborations between basic and clinical researchers and ultimately paves the way for improved outcomes for sepsis patients.

### Experimental design

- Experimental sepsis research must be designed, conducted and reported in such a way to minimise bias and to maximise the potential for replication.

### 3Rs importance of harm:benefit assessement

- Harm:benefit impact assessment and full scientific justification should be at the forefront of any new studies involving research animals.
- Harm relates to welfare experience of animal whilst benefit relates to value of scientific data. Data should be interpreted in the context of what the model can deliver, avoiding over-translation.

### 3Rs impact of construct validity models

- Focussing on construct rather than face validity, has the same potential to increase clinical translation, but with reduced levels of animal suffering.

### Mechanistic models of inflammatory and immune pathways and enhancing the translational gap

- As our knowledge of the innate immune responses in sepsis increases, new therapeutic targets and new potential biomarkers to measure sepsis progression and response to therapy are emerging. These include metabolic markers that underlie trained immunity and immune cell activation and biomarkers of inflammasome activation which could be mechanistically investigated in preclinical models. As tools and technologies for systems biology and high-throughput screening advance, there can be an increased use of humans and human-derived tissue for sepsis research models. Development of more sophisticated models will enable the identification of new immune mechanisms that can be translated into new diagnostic and therapeutic practices.

### Cardiovascular monitoring strategies that support the 3Rs

- Animal models may still be required to validate safety and efficacy of new therapeutic entities that target the cardiovascular system. Several minimally invasive technologies and *in vitro* models are available which can help reduce, refine and replace animal experiments. Real-time monitoring technologies can encourage use of scientifically valuable, humane endpoints, whilst avoiding mortality endpoints. Such endpoints may be incorporated into future clinical studies.

### Mimicking the clinical setting where regulators require sepsis to be modelled

- Current preclinical approaches are not delivering for sepsis patients. We propose the concept that a single animal model could be the ‘gold standard’ predictor of clinical success should be abandoned. Mechanistic models, derived from human clinical data, with a focus on target engagement rather than clinical phenotype should be more predictive. If disease (face validity) models are required, these should mimic human clinical ICU settings.

### Top-down approach to sepsis research

- A shift of focus, from animal-based approaches to one that places more emphasis on human clinical data will benefit more patients in the short term and identify new therapeutic targets for drug development in the longer term.

## Abbreviations

ASC: apoptosis-associated speck-like protein
CLP: caecal ligation and puncture
CRP: C-reactive protein
ECG: electrocardiography
GWAS: genome-wide association study
HIF: hypoxia-inducible factor
HPA: hypothalamic pituitary axis
ICU: intensive care unit
IL: interleukin
LPS: lipopolysaccharide
NLRP3: NOD-, LRR- and pyrin domain- containing protein 3
RCT: randomised controlled trial
T2DM: type 2 diabetes mellitus
T-reg: regulatory T cell
TLR: toll-like receptor
3Rs: replacement, reduction and refinement
